# Prospective evaluation of bone markers, parathormone and 1,25-(OH)_2 _vitamin D in HIV-positive patients after the initiation of tenofovir/emtricitabine with atazanavir/ritonavir or efavirenz

**DOI:** 10.1186/1471-2334-12-38

**Published:** 2012-02-14

**Authors:** Emanuele Focà, Davide Motta, Marco Borderi, Daria Gotti, Laura Albini, Alessandra Calabresi, Ilaria Izzo, Rita Bellagamba, Pasquale Narciso, Laura Sighinolfi, Alberto Clò, Davide Gibellini, Eugenia Quiros-Roldan, Nigritella Brianese, Bruno Mario Cesana, Maria Carla Re, Carlo Torti

**Affiliations:** 1 Infectious Diseases Department, University of Brescia, Brescia, Italy; 2 Infectious Diseases Section, University of Bologna, Bologna, Italy; 3 National Institute of Infectious Diseases, Rome, Italy; 4 Infectious Diseases Department, S. Anna Hospital, Ferrara, Italy; 5 Microbiology Section, University of Bologna, Bologna, Italy; 6 Medical Statistics and Biometry Section, University of Brescia, Brescia, Italy; 7 Institute of Infectious and Tropical Diseases, University of Brescia, School of Medicine, P.le Spedali Civili, 1, 25123 Brescia, Italy

**Keywords:** HIV, Antiretroviral therapy, Bone turnover, Osteoporosis, Vitamin D

## Abstract

**Background:**

Increased risk of fractures and osteoporosis have been associated with the use of antiretroviral drugs. There is a paucity of prospective evaluations of bone markers after the initiation of drugs currently recommended to treat HIV infection and results on the evolution of these markers are conflicting. Lastly, the effect of tenofovir on 1,25-(OH)_2 _vitamin D is uncertain.

**Methods:**

We performed a prospective study on the evolution of bone markers, parathormone and 1,25-(OH)_2 _vitamin D before and after standard antiretroviral regimens. This was a sub-study of a trial conducted in antiretroviral-naïve patients randomized to tenofovir + emtricitabine in combination with either atazanavir/ritonavir (ATV/r) or efavirenz (EFV). Follow-up lasted 48 weeks. The following bone markers were analyzed: C-terminal cross-laps (CTx), osteocalcin (OC), osteoprotegerin (OPG), and receptor activator of nuclear factor κB ligand (RANKL). Mixed-factorial analysis of variance with random-coefficient general linear model was used to compare their trends over time and linear multivariable regression was performed with a backward selection method to assess predictors of their variations from baseline to week 48. Trends of parathormone and 1,25-(OH)_2 _vitamin D were also evaluated.

**Results:**

Seventy-five patients were studied: 33 received EFV and 42 ATV/r. Significant increases were found for all markers except for RANKL. There was a significant direct association between CTx and OC increases. Multivariable analysis showed that higher glomerular filtration rate (estimated through cystatin C clearance) predicted greater OPG increase, while older age, higher HIV RNA at baseline and use of ATV/r predicted greater CTx increase. A significant increase of parathormone accompanied the evolution of the study markers. 1,25-(OH)_2 _vitamin D remained stable, though a seasonality variation was demonstrated.

**Conclusions:**

These data demonstrate CTx increase (bone resorption marker) corresponding to OC increase (bone formation marker) early upon HAART initiation. Moreover, predictors of bone marker increases have been suggested, possibly indicating that a stricter monitoring of bone health and pro-active interventions are needed in older patients, those with higher HIV RNA, prescribed ATV/r rather than EFV, and with decreased renal function at baseline. Further studies are needed to clarify the mechanisms responsible for up-regulation of bone turnover markers, as well as to understand if and what markers are best correlated or predictive of pathological fractures.

## Background

Highly active antiretroviral therapy (HAART) consisting of 2 nucleoside reverse transcriptase inhibitors (NRTI) (i.e., nucleoside backbone) combined with either a protease inhibitor (PI) or a non nucleoside reverse transcriptase inhibitor (NNRTI) (i.e., anchor drugs) have dramatically reduced opportunistic infections and death in HIV infected patients. However, despite these benefits, several co-morbidities, including osteoporosis [1], have emerged. In HIV-infected patients, osteoporosis is present with an overall prevalence of about 15%, and fragility fractures are becoming more frequent than in the general population [2,3]. Therefore, screening and prevention strategies should be implemented to diagnose bone alterations.

Several biochemical markers of bone turnover have been proposed to detect early-onset modifications in bone formation (such as osteocalcin, OC) or bone resorption (C-terminal cross-laps, CTx) [4]. These markers could also help to evaluate the effectiveness of anti-osteoporotic therapy [5]. Moreover, although osteoprotegerin (OPG) and receptor activator of nuclear factor κB ligand (RANKL) are not proper osteoporotic markers since their levels in blood do not entirely reflect those present inside bone, they are considered major regulators of bone remodeling, so we studied them to explore osteoclastogenesis [6].

The mechanisms responsible for osteopenia and osteoporosis are uncertain but exposure to certain antiretroviral drugs (in particular a NRTI: tenofovir--TDF--and the PI class), aging, HIV by itself, parathormone (PTH) increase, and vitamin D deficiency may be implicated [7-10].

Discordant results regarding levels and evolution of bone turnover markers in HIV patients on HAART have been demonstrated [11-16]. Moreover there is a paucity of data on predictors of changes in these parameters [11-16]. In particular, discordant results regarding the possible impact of different types of antiretroviral drugs on changes in bone turnover markers currently exist.

Therefore, the main objective of this study was to describe the evolution of bone markers in HIV infected patients starting the HAART regimens currently recommended [17,18]. We also aimed to evaluate possible predictors of the evolution of these markers (including class of antiretroviral therapy used as anchor).

## Methods

This was a sub-study of a pilot open-label, multicenter, randomized controlled trial conducted in 91 patients without chronic kidney diseases to evaluate renal function after TDF + emtricitabine (FTC) plus either atazanavir boosted by ritonavir (ATV/r) or efavirenz (EFV) in HIV-infected patients naive to antiretroviral therapy (EudraCT number: 2007-007934-21) [19].

Seventy-five patients who remained in the randomized treatment arms were included. Baseline data included clinical history, CD4+ T-cell count, HIV RNA (branched chain DNA-enhanced label amplification assay, Quantuplex 2-0; Chiron, with a 50 copies/mL cut-off) and HBV/HCV serostatus. Clinical examination, HIV RNA, CD4+ T-cell count and routine laboratory tests (including creatinine and electrolytes) were assessed during follow-up. Plasma samples were taken and stored at -80°C until they were de-frozen for the present analyses.

Enzyme immunoassay techniques were used for all markers: OC (inter-assay and intra-assay coefficients of variation--CV--4.0% and 1.8%, respectively), CTx (inter-assay CV 7.7%, intra-assay CV 2.2%) and 1,25-(OH)_2 _vitamin D (inter-assay CV 6.6%, intra-assay CV 5.9%) were determined with EIA kits by IDS (Boldon, UK); OPG (inter-assay CV 6.9%, intra-assay CV 3.8%) and RANK-L (inter-assay CV 7.5%, intra-assay CV 5.7%) were determined with EIA kits by Peprotech (London, UK); lastly, PTH was determined with a DIAsource ImmnoAssays S.A. EIA kit (Nivelles, Belgium; inter-assay CV 5.0%, intra-assay CV 1.6%). Manufacturers' procedures were followed as indicated in the instruction manuals. All analyses were performed at the Microbiology Section, University of Bologna, Bologna, Italy. The following ranges of normality were considered: OC in women 8.4-33.9 ng/mL, in men 9.6-40.8 ng/mL; CTx in women 0.112-0.738 ng/mL, in men 0.115-0.748 ng/mL. Hyperparathyroidism cut-off was 65 pg/ml, and vitamin D deficiency was defined as < 11 pg/mL. Clinically validated ranges of normality for OPG and RANKL currently do not exist.

Glomerular filtration rate (GFR) was estimated by the CKD-EPI formulae since they are more suitable for patients without chronic kidney disease using both creatinine [20] and cystatin C [21] values, corrected for body surface area (BSA) by the DuBois method [22].

The study was conducted in accordance with good clinical practice (ICH-E6) [23]. The protocol and amendments were approved by the institutional review boards and the patients gave written informed consent before screening at each study site. The enrolment period lasted from June 2007 to April 2009.

### Statistical analysis

Descriptive statistics was calculated for quantitative variables (mean, standard deviation--SD, median, minimum and maximum) and qualitative variables (absolute and percentage frequencies). Ninety-five percent confidence intervals (95%CI) were calculated as appropriate. The unpaired Student's t test was used to compare quantitative variables (Wilcoxon's Rank Sum test for variables that were not Gaussianly distributed) and the χ^2 ^test (Fisher's exact test) for qualitative variables.

The overall time-variation of the markers was evaluated by a mixed-factorial analysis of variance (ANOVA) with random-coefficient general linear model.

Differences between week-48 and baseline values were calculated as delta (Δ_[wk 48-bl]_) for each marker. Pearson's correlation coefficient was used to assess the relationship of the markers' Δ_[wk 48-bl] _between each other.

Demographical, clinical and laboratory characteristics at baseline (listed in Table 1), as well as variations of eGFR, PTH, 1,25-(OH)_2 _vitamin D and bone turnover markers from baseline to week 48 were tested at univariate analysis for their associations with the outcomes (Δ_[wk 48-bl] _for OPG, CTx, or OC). Factors that resulted associated with the outcomes at univariate analysis with a *p*-value < 0.10, as well as factors of clinical importance, such as antiretroviral treatment received, were included in a multivariable regression model with backward selection in order to obtain the most parsimonious set of the variables that predicted Δ_[wk 48-bl] _for each of the bone turnover markers as outcome measures.

**Table 1:** Patients' characteristics at baseline

Variable	N(%)
Age, years [mean (SD)]	41.6 (11.9)

Males	61 (81.3)

Risk factor IVDU	6 (8)

BMI (< 25 Kg/m^2^)	39 (52.7)

Viral load > 100.000 copies/mL	26 (34.7)

CD4 T cell count < 200 cells/mm^3^	17 (22.7)

CD4/CD8 ratio < 0.4	42 (56)

Cigarette smokers	35 (46.7)

HCV Ab positive	11 (14.7)

CDC 93 clinical class A	46 (65.7)

HAART:	

• TDF/FTC + EFV	33 (44)

• TDF/FTC + ATV/r	42 (56)

Phosphoraemia < 2.7 mg/dL	19 (25.3)

1,25 (OH)2 Vitamin D < 11 pg/mL	19 (25.3)

Hyperparathyroidism (> 65 pg/mL)	4 (5.3)

eGFR creatinine * < 90 mL/min	26 (34.6)

eGFR cystatin C * < 90 mL/min	15 (20.5)

OC, ng/mL [mean (SD)]	19.86 (10)

CTx, ng/mL [mean (SD)]	0.43 (0.26)

OPG, ng/mL [mean (SD)]	0.83 (0.41)

*N *number, *SD *standard deviation, *IVDU *intra-venous drug users, *BMI *body mass index, *HAART *highly active antiretroviral therapy, *TDF *tenofovir, *FTC *emtricitabine, *EFV *efavirenz, *ATV/r *atazanavir/ritonavir, *eGFR *estimated glomerular filtration rate, *OC *osteocalcin, *CTx *C-terminal cross-laps, *OPG *osteoprotegerin

RANKL at baseline was not dosable in 86% of patients

To account for possible seasonal variations in 1,25-(OH)_2 _vitamin D, we stratified patients into two groups according to the month of HAART initiation (*sunny-period *between May and October and *not-sunny-period *between November and April). All patients resided in a region between 41° and 45° latitude North; these periods were selected because 1,25-(OH)_2 _vitamin D production is impossible during the *not-sunny *months owing to the solar zenith angle, which influences the incident UVB radiation [24].

All analyses were performed using the statistical software package SAS^® ^(SAS Istitute Inc., Cary NC, USA) version 9.13. All *p *values were considered significant if < 0.05 or according to Bonferroni's significance adjustment. With 38 patients in each group, there is a power of 0.80 of demonstrating an effect size of about 0.65 at an unpaired Student's t test with a significance level of 0.05 (two tailed).

## Results

### Patients' characteristics at baseline

Among 91 patients enrolled in the trial, 75 remained on follow-up with the randomized HAART regimens and had plasma samples available, so they were included in this sub-study (33/75 receiving EFV and 42/75 ATV/r). All patients achieved HIV RNA < 50 copies/mL by week 24 and maintained virological success up to the end of the follow-up. CD4+ T-cell count increased from baseline to week 48 by a mean of 202 (SD: 172) cells/mm^3^. One patient withdrew the informed consent after week 48 for the main trial but not for this sub-study. Sixteen patients discontinued the study, none of them for renal failure or bone alterations.

Patients' characteristics are shown in Table 1. Most patients were males and acquired HIV infection through sexual intercourse, 77.3% had CD4+ T-cell count > 200/mm^3 ^and more than half had not experienced any HIV-related events. A quarter of patients (19/75) showed hypophosphoremia (< 2.7 mg/dL) and a considerable proportion (22.6%) of patients had serious 1,25-(OH)_2 _vitamin D deficiency (i.e., < 11 pg/mL) at baseline. Mean PTH at baseline was 31.04 (SD: 17.7) pg/mL; considering 65 pg/mL as a cut-off, 4/75 (5.3%) patients had hyperparathyroidism. Most patients initiated antiretroviral therapy with normal kidney function calculated using either creatinine or cystatin C clearance. These characteristics were similar to those of the main trial including 91 patients [19].

All patients had normal serum calcium levels at baseline. In 30 patients in whom FRAX™ scores [25] could be calculated because age was > 40 years the mean score was 3.12 (SD: 0.87).

Values of bone turnover markers at baseline are shown in Table 1. Of note, RANKL was undetectable (< 0.0063 pg/mL) in 86% patients. As for the other markers, we found that, at baseline, 2/75 patients exceeded the OC cut-off, while 8/75 (10.6%) exceeded the CTx cut-off.

### Trends of the bone turnover markers

**Figure 1:**
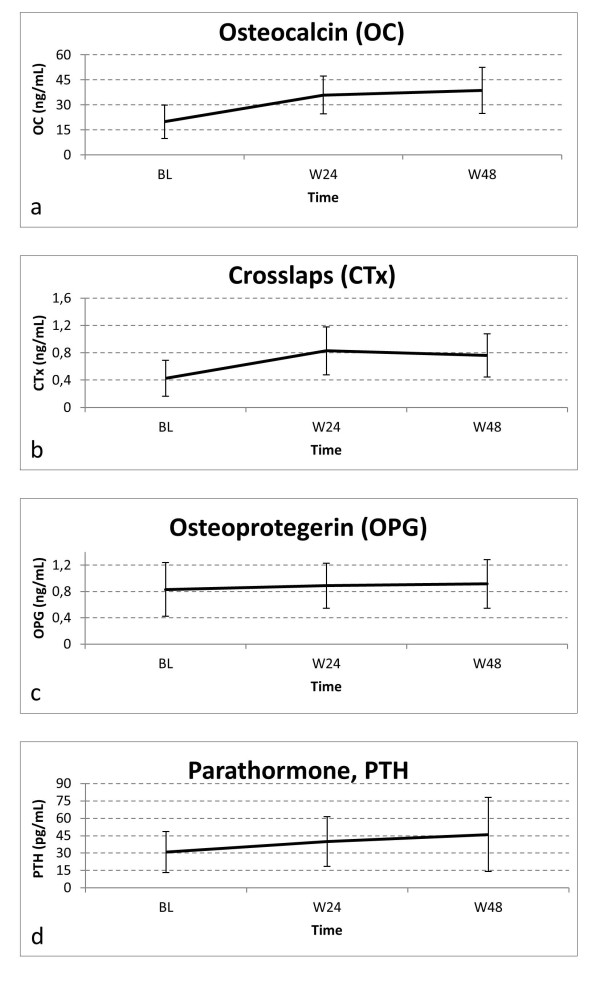
**Trends in bone turnover markers and PTH**. Trends of mean (SD) in bone turnover markers and PTH: **a**) Osteocalcin Δ_[48-bl] _= 18.78 ng/mL *p *< 0.0001; **b**) CTx Δ_[48-bl] _= 0.33 ng/mL, *p *< 0.0001; **c**) OPG Δ_[48-bl] _0.09 ng/mL, *p *= 0.0432; **d**) PTH Δ_[48-bl] _= 15.05 ng/mL, *p *= 0.0002.

There was a significant increase in bone markers during the follow-up (Figure 1a, b, c). Mean Δ_[wk 48-bl] _were as follows: 18.78 (SD: 15.5) ng/mL for OC, 0.33 (SD: 0.31) ng/mL for CTx, and 0.09 (SD: 0.36) ng/mL for OPG. Up to week 48, 32/75 (42.7%) patients for OC and 24/75 (32%) for CTx exceeded the respective cut-offs of normality. Therefore, with respect to baseline, there were significant increases in proportion of patients above the cut-offs (*p *< 0.01).

RANKL remained or became undetectable in 87% patients throughout the follow-up. Linear correlation analysis to explore relationship between Δ_[wk 48-bl] _of the study markers showed that OC increased with increasing CTx (correlation coefficient: 0.26; *p *= 0.026), while no other significant correlations between each other were found.

### Trends of PTH and 1,25-(OH)_2 _vitamin D

PTH increased significantly by a mean (Δ_[wk 48-bl]_) of 15.05 (SD: 35.76) pg/mL (Figure 1d). At week 48 there were 9 (12%) patients with an elevated PTH.

**Figure 2:**
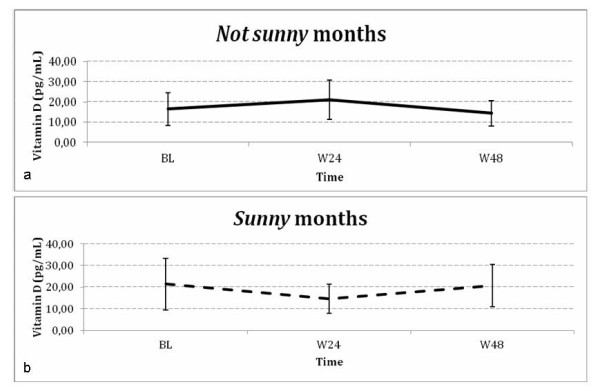
**Seasonality of 1,25-(OH)_2 _vitamin D levels**. Patterns of 1,25-(OH)_2 _vitamin D levels overtime related to seasonality: **a**) *Sunny *months (between May and October)\**b**) *Not sunny *months (between November and April). No significant differences between the two groups were found in Δ_[48-bl] _1,25-(OH)_2 _vitamin D (*p *= 0.2364), but different patterns of vitamin D related to seasonality were found (*p *< 0.0001).

There were no significant variations in 1,25-(OH)_2 _vitamin D levels in the overall population and no significant differences in Δ_[wk 48-bl] _were found by period of treatment initiation (*sunny*--versus--*not sunny*) (*p *= 0.2364); however, as shown in Figure 2, different patterns related to seasonality were found because there was a transient increase at week 24 in patients who started therapy in the *not-sunny *period, while a decrease in those who started in the *sunny period *was found (*p *< 0.0001). At week 48 patients with a deficit of vitamin D were 19 (25.3%). None of the patients received vitamin D supplementation during the study.

### Predictors of increase in bone turnover markers

At univariate analysis we found significant associations of greater OPG Δ_[wk 48-bl] _with higher CD4+/CD8+ T-cell ratio (r^2 ^= 0.06, *p *= 0.03), and with greater eGFR estimated by CKD-EPI based on cystatin C values at baseline (r^2 ^= 0.06, *p *= 0.03), but only the latter maintained a borderline significance at multivariable model (Table 2), indicating that there was a greater increase in OPG (mean: 0.21 ng/mL) in patients with better renal function at baseline.

**Table 2:** multivariable regression analysis

Variable	β	SE	*p*
	*Δ CTx*		

ATV/r vs. EFV	-0.22	0.06	0.002

Age (for one year older)	0.01	0.01	0.009

Log_10 _HIV RNA (for 1 log_10 _higher)	0.07	0.02	0.002

	*Δ OPG*		

eGFR cystatin C (< 90 vs. > 90 L/min)	0.21	0.1	0.045

*β *partial regression coefficient, *SE *standard error, *EFV *efavirenz, *ATV/r *atazanavir/ritonavir, *eGFR *estimated glomerular filtration rate, *CTx *C-terminal cross-laps, *OPG *osteoprotegerin.

CTx Δ_[wk 48-bl] _was associated at univariate analysis with increasing OC Δ_[wk 48-bl] _(r^2 ^= 0.07, *p *= 0.026), lower CD4+/CD8+ T-cell ratio at baseline (r^2 ^= 0.07, *p *= 0.038), smaller eGFR _Creatinine _Δ_[wk 48-bl] _(r^2 ^= 0.1, *p *= 0.009), older age (r^2 ^= 0.05, *p *= 0.05), and higher HIV RNA at baseline (r^2 ^= 0.04, *p *= 0.06). At multivariable analysis including treatment group as clinically significant variable, older age, higher HIV RNA at baseline, and the use of ATV/r were significantly associated with CTx increase. Particularly, as for treatment received, patients prescribed ATV/r had a mean of 0.22 ng/mL CTx increase, independently from the other two factors.

For OC Δ_[wk 48-bl]_, no significant associations besides that with CTx Δ_[wk 48-bl] _were found.

## Discussion

There is a paucity of data on bone turnover markers in HIV-infected patients after HAART. Some studies demonstrated an increase in OC and CTx [14,15] and these increases correlated with reduction of bone mineral density (BMD) [15]. By contrast, other authors found significant decreased levels of OC in patients treated with HAART [12,13]. Our data confirm a parallel increase of soluble markers indicating bone formation (OC) and resorption (CTx) upon initiation of HAART and this effect appeared to be maximal at week 24.

We found a significant increase of OPG after HAART, differently from the study by Brown et al. [14] who found a decrease of this marker. The authors hypothesized that decreased inflammation after HAART could reduce OPG. In the present study, inflammation was not assessed, but all patients achieved sustained undetectable HIV RNA (the main driver of inflammation), therefore other factors may be implicated.

Higher eGFR at baseline (measured by cystatin C using the CKD-EPI formula) predicted OPG increase from baseline to week 48. Glomerular filtration rate estimated through cystatin C may be a reliable method to assess renal function in HIV infected patients because it is less influenced by muscle mass and liver function than the methods based on creatinine [26,27]. Therefore, our data suggest that patients with better eGFR are protected since OPG by itself is known to counteract osteoclastic activation by antagonism to RANKL [28]. However, since cystatin C is increased by inflammation [29,30], it has to be seen whether the pro-inflammatory status at baseline or the actual kidney damage is a better predictor OPG evolution.

Older age and increasing HIV RNA at baseline were independently predictive of CTx increase during the follow-up. Since the increase in CD4+ T-cells is greater in patients with higher HIV RNA at baseline [31,32] the correlation between higher HIV RNA and CTx increase could be mediated by immune-reconstitution, as recently suggested [33]. However, no significant correlation between Δ_[wk 48-bl] _of the CD4+ T-cell count and CTx increase was found in our study (data not shown), and this seems to contradict the previous hypothesis [33]. Therefore, greater increase of CTx could be due to the fact that bone health is already impaired in older patients and in those with higher HIV RNA due to a damaging effect of HIV on bone health [34,35]. However, the underlying mechanisms should be investigated.

Notably, the use of ATV/r was associated with greater CTx increase in our study. Brown et al. [14] found that OC increase, but not CTx increase, was associated with PI use. Notwithstanding this apparent inconsistency, both studies suggest that PIs are associated with up-regulation of bone remodeling. Although we could not provide reliable explanations, McComsey et al. [36] already demonstrated a deeper impact of ATV/r on lumbar vertebral BMD than EFV when co-administered with TDF. Our results are in line with--and may explain--this observation.

We found a significant PTH increase after HAART, confirming previous studies [8,37,38]. This increase could be due to low 25(OH) vitamin D levels. However, we did not evaluate 25(OH) vitamin D, so this hypothesis could not be proven. Rather, we were interested in 1,25-(OH)_2 _vitamin D in order to assess the availability of the final product exerting biological activity. We found that 1,25-(OH)_2 _vitamin D remained stable, while in other studies TDF seemed to increase it [11,39]. This stability was observed independently from variations due to seasonality, a finding that was not confirmed by others [40]. Therefore, the effect of TDF on 1,25 (OH)_2 _vitamin D needs further considerations.

This study suffers from several limitations. First of all, BMD was not assessed; therefore the early activation of bone markers could not be coupled with BMD reduction. Second, 1,25-(OH)_2 _vitamin D levels are unstable and its values are of difficult interpretation [41]. Third, our study is descriptive, so we did not provide any data on possible mechanisms. For instance, it is impossible to infer whether the increase in bone markers was due to PTH increase or to a direct effect of antiretroviral drugs on bone metabolism. Fourth, the number of patients was small, but the study provided statistically significant results. Lastly, we studied a sub-group of patients from the randomized cohort based on the availability of stored samples and maintenance of the initial HAART regimen. Although we could not exclude a selection bias, this is unlikely because no patients dropped out of the study for bone complications and they were not systematically selected for inclusion in the study. Notwithstanding these limitations we believe that our results add interesting information given the paucity of the current literature data.

## Conclusions

The rapid and significant increase in bone markers observed in the present study reinforce that a strict monitoring is necessary to detect early signs of bone damage as already stated by recent guidelines [18]. Moreover, we identified possible predictors of bone turnover changes (such as age, HIV RNA, ATV/r co-administration and kidney function) which may help identify most-at-risk patients. Since a correlation between increase of soluble bone markers and reduction of BMD has already been found, our results can be clinically significant. Moreover, it has been hypothesized that bone turnover markers may offer additional or complementary information with respect to BMD assessment through DXA scan [4]. However, more powerful studies are needed to correlate changes in bone turnover markers with bone mineral density and risk of fractures, so as to understand if these markers (and what) should be monitored in clinical practice [42]. Our results, however, suggest that OC and CTx are good candidates.

## Competing interests

Some authors (EF, MB, EQR, MCR and CT) received grants from several Pharmaceutical Companies for participating to advisory board and scientific conferences but the received supports did not influence the content of this paper. The remaining authors declare that they have no competing interests.

## Authors' contributions

*Study concept and design: *CT, EF, DM. *Acquisition of data: *ACa, II, RB, PN, LS, EQR, NB. *Laboratory analyses*: ACl, MCR, DG. *Analysis and interpretation of data: *CT, EF, DM, BMC. *Drafting of the manuscript: *CT, EF, DM. *Critical revision of the manuscript for important intellectual content: *MB, II, RB, PN, LS, EQR, DG, NB, LA, BMC. *Statistical analysis: *BMC. All authors read and approved the final manuscript.

## Pre-publication history

The pre-publication history for this paper can be accessed here:

http://www.biomedcentral.com/1471-2334/12/38/prepub
